# Delaying the International Spread of Pandemic Influenza

**DOI:** 10.1371/journal.pmed.0030212

**Published:** 2006-05-02

**Authors:** Ben S Cooper, Richard J Pitman, W. John Edmunds, Nigel J Gay

**Affiliations:** **1**Statistics, Modelling, and Bioinformatics Department, Centre for Infections, Health Protection Agency, London, United Kingdom; Mexican National Institutes of HealthMexico

## Abstract

**Background:**

The recent emergence of hypervirulent subtypes of avian influenza has underlined the potentially devastating effects of pandemic influenza. Were such a virus to acquire the ability to spread efficiently between humans, control would almost certainly be hampered by limited vaccine supplies unless global spread could be substantially delayed. Moreover, the large increases that have occurred in international air travel might be expected to lead to more rapid global dissemination than in previous pandemics.

**Methods and Findings:**

To evaluate the potential of local control measures and travel restrictions to impede global dissemination, we developed stochastic models of the international spread of influenza based on extensions of coupled epidemic transmission models. These models have been shown to be capable of accurately forecasting local and global spread of epidemic and pandemic influenza. We show that under most scenarios restrictions on air travel are likely to be of surprisingly little value in delaying epidemics, unless almost all travel ceases very soon after epidemics are detected.

**Conclusions:**

Interventions to reduce local transmission of influenza are likely to be more effective at reducing the rate of global spread and less vulnerable to implementation delays than air travel restrictions. Nevertheless, under the most plausible scenarios, achievable delays are small compared with the time needed to accumulate substantial vaccine stocks.

## Abstract 2

Air travel might contribute to the spread of influenza in future pandemics. However, this modelling study concluded that restrictions on air travel would not provide effective control.

## Introduction

The scale of threat posed by hypervirulent avian influenza subtypes [1,2], and the memory of the 20–100 million who died in the 1918 pandemic [3,4], warrant consideration of large-scale, concerted, and potentially highly disruptive control measures [5]. Were such a virus to acquire the ability to spread efficiently between humans, control would almost certainly be hampered by limited vaccine supplies [6]. Interventions able to substantially impede global spread, by providing time for vaccine stocks to accumulate, could have profound public health benefits.

Border controls and World Health Organization travel advisories formed central and sometimes controversial components of the control efforts during the severe acute respiratory syndrome (SARS) epidemic [7,8], and travel restriction is thought likely to occur during an influenza pandemic (although enforcement is currently considered by the World Health Organization to be impractical in most countries) [9]. In the absence of sufficient vaccine stocks, other control measures such as the use of antiviral agents could also be used [10,11]. Ideally, such measures would reduce the average number of secondary cases caused by each primary case (the effective reproduction number, *R*_t_) to below one, making sustained transmission impossible. This happened during the SARS epidemic, where isolation, quarantine, and behaviour change were able to bring about control [12]. The much shorter serial interval for influenza makes the chances for early epidemic termination much lower [13]. The main value of interventions is more likely to be in reducing the incidence and slowing the rate of spread of the virus.

To evaluate the potential of travel restriction and local control measures to impede global dissemination we developed a stochastic (i.e., probabilistic) model of the international spread of influenza based on extensions of coupled deterministic epidemic transmission models [14–19]. This class of models has been shown to be capable of accurately forecasting local and global spread of epidemic and pandemic influenza [14–19] and accounting for the global distribution of other pathogens [20,21], but has not previously been used to assess the impact of travel restrictions or other control options for pandemic influenza.

## Methods

**Figure 1:**
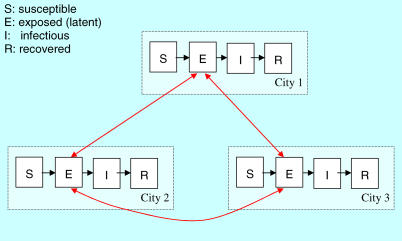
Schematic Illustration of Model Structure

We used a metapopulation model that consists of a set of coupled dynamic epidemic transmission models (Figure 1). Each component model represents one city and tracks the progression of individuals through four classes: susceptible to infection (S); exposed to the virus but not yet infectious (E); infectious (I); and recovered and no longer susceptible (R). We assumed that infectiousness coincides with disease onset and that infectious cases do not travel.

Previous work has used deterministic approximations to study the evolution of this system [14–19]. With this approach the first case in each city (except the originating city) was assumed to occur only when the average number of incubating cases arriving from other cities exceeded one, an approximation that will artificially slow the rate of spread between cities. Our stochastic model, which has a similar underlying structure to its deterministic counterpart, avoids this distortion and uses probabilistic transitions to capture the inherent uncertainty in the course of the epidemic. This is more appropriate than the deterministic approaches previously adopted, because chance effects dominate in the early stages of the epidemic in each city and in the seeding of each city's epidemic. As well as providing greater realism, this approach allowed us to quantify the uncertainty in model predictions due to demographic stochasticity. Thus, rather than assuming that each city's epidemic starts at a determined time, we assumed that the initiation of an epidemic depends on the timing of a sequence of chance events: a person incubating the virus must board a plane; that person must infect others in the destination city; some of those others must cause further transmission, and so on. Each time the model is run, even with identical starting conditions and parameter values, a different answer is obtained. This stochastic model was used to estimate key parameters using data from the 1968–1969 (1968/9) influenza pandemic and to evaluate the impact of interventions using contemporary demographic and transport data. Mathematical details of the model are provided in Protocol S1.

Coupling between cities was estimated using data from the International Air Transport Association for 2002 that gives the number of seats on flights between 105 cities, including the 100 with the highest number of international scheduled passengers and all 52 used in the 1968/9 data. City sizes were taken from the United Nations urban agglomeration data (available at: http://unstats.un.org/unsd/demographic/sconcerns/densurb/urban.aspx). When fitting models to 1968/9 data, air transport data, sizes of urban agglomerations, and influenza data were taken from a previous study [14,15].

To select from variants of the basic model, we compared deterministic and stochastic model fits to data from the 1968/9 pandemic by choosing parameters to minimize the sum of squared deviations (SSQ) between times of observed and predicted peaks (this contrasts with previous work that aimed to forecast the pandemic, and therefore based parameter estimates only on data from the first affected city [14]). A major strength of our approach is that the observed time of an epidemic peak should be unaffected even by large between-country variation in influenza reporting rates. The predicted epidemic peak for a given city was defined as the day on which the highest number of infected people first developed symptoms. When a city had more than one observed peak in the 1968/9 data, only the first peak was used. We used the statistic SSQ(*m*)/(*n* − 2*m*) to compare model fits [22], where *m* is the number of fitted parameters (between one and four), and *n* the number of fitted data points (the number of cities with observed and predicted epidemic peak times). This formula selects models that are parsimonious and fit the data well. The full stochastic model was then used to estimate parameters for the best-fitting model by choosing parameters that minimised the mean SSQ from ten simulation runs for each combination of parameter values.

We evaluated models with sine wave, square wave, and no seasonal variation in transmission parameters for cities outside the tropics. In the sine wave formulation, the peak transmissibility occurred on the shortest day in each hemisphere, while in the square wave formulation, peak transmissibility lasted 6 mo, also centred on the winter solstice. We also considered model formulations where the transmission parameter in the tropics was taken as the maximum, minimum, and mean over 1 y of that outside the tropics. For some parameter values, the model predicted no epidemic peaks in some cities for which an epidemic peak was in fact recorded. When fitting the models we penalized these regions of parameter space by arbitrarily assigning a deviation between model and data of 500 d.

### Interventions

We used the stochastic model to consider the effects of (i) reducing local transmission (this simulates the effects of isolation, behaviour changes, antiviral use, or other measures that may reduce the average number of secondary cases produced by one primary case); and (ii) restricting travel to and from affected cities. We assessed the ability of these measures to delay epidemics in individual cities. We considered only major epidemics, which we defined as those peaking with at least one case per 10,000 people per day. We assumed that measures were introduced only after the first 100 symptomatic cases in each city except the originating city, for which 1,000 cases were required, although we also evaluated the sensitivity of the results to these assumptions.

We considered a number of other scenarios to assess the sensitivity of the results to the most important unknowns: patterns of seasonal variation in influenza transmission; variation in transmissibility between tropical and temperate regions; the proportion of individuals initially susceptible to the virus; the basic reproduction number, *R*_0_ (defined as the mean number of secondary cases in a local and susceptible population caused by the introduction of one primary case); the distribution of the infectious period; the city in which the pandemic begins; and the date on which the virus first begins to spread.

## Results

**Figure 2:**
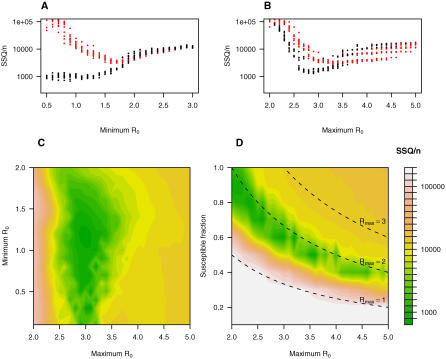
Model Fitting and Comparison by Sum of Squared Deviations (A and B) SSQ/*n* values for ten runs with each combination of parameter values for two models: model 1 (black), in which transmissibility in the tropics is constant and taken as the north/south maximum; and model 2 (red), in which it is held at the north/south mean. *n* is the number of cities with observed peaks. The seasonal maximum *R*_0_ (*R*_0,max_) is held constant at 3.0 in (A), and the seasonal minimum *R*_0_ (*R*_0,min_) is held constant at 1.6 (the value giving the minimum SSQ/n for model 2) in (B). In both cases, the fraction of the population initially susceptible was fixed at 0.6. (C and D) SSQ/*n* values shown are the means of ten runs of the stochastic model for each combination of parameter values. Regions of parameter space supported by the data appear in green (darker green indicates better fit). A constant susceptible fraction of 0.6 is assumed in (C). *R*_0,min_ held constant at 1.2 in (D); broken lines indicate regions of the parameter space with constant effective reproduction numbers, *R_max_* (where *R_max_* = *R_0,max_*× susceptible fraction).

Amongst the model variants considered, the best fit to data from the 1968/9 pandemic was achieved when transmissibility varied sinusoidally in temperate regions and was constant and equal to the north/south maximum in the tropics. We used this model to estimate key parameters using 1968/9 data, and to evaluate the impact of interventions. Models without seasonal forcing terms gave poor fits to data and could not account for the large differences in epidemic timing between cities in the north and south temperate regions. Models in which transmissibility in the tropics was set to the north/south mean also performed surprisingly poorly, with best-fit SSQs approximately three times greater than those obtained when transmissibility in the tropics was set to the north/south maximum (Figure 2A and 2B). Less surprisingly, models that assumed all cities were equally connected by air travel (but with the same total volume of air traffic) also performed poorly, with best-fit SSQs about twice as large as those from the models that used the air travel data. Previous work with the deterministic version of the model has assumed a square wave variation in transmissibility, assigning transmission outside the influenza season to be one-tenth of the value during the season [14]. We found the fit to data under this assumption to be substantially poorer compared with models in which maximum and minimum seasonal transmission parameters were both estimated.

An exploration of the parameter space for the best-fit model showed that, assuming 60% of the population to be initially susceptible (the approximate value estimated previously [14]), maximum *R*_0_ values (*R*_0,max_) ranging from about 2.5 to 3.5 gave the best fits to data, while minimum *R*_0_ values (*R*_0,min_) between about 0.5 and 1.5 had the most support (Figure 2C). The maximum *R*_0_ value and the fraction initially susceptible could not be identified simultaneously: A high value of one implied a low value of the other (Figure 2D). However, the initial maximum *effective* reproduction number, *R*_max_ (equal to the product of the two and giving the average number of secondary cases produced by one primary case in an actual population, accounting for immunity) was well defined, with only a narrow range of values between about 1.5 and 2.2 supported by the data. This result is consistent with other estimates from influenza pandemics [14,23]. We therefore took as our baseline scenario an *R*_0,max_ value of 3 and an *R*_0,min_ of 1.2, assumed 60% of the population to be initially susceptible, and used a model in which the *R*_0_ value varied sinusoidally and peaked in midwinter, and in which the pandemic originated in Hong Kong on 1 June.

**Figure 3:**
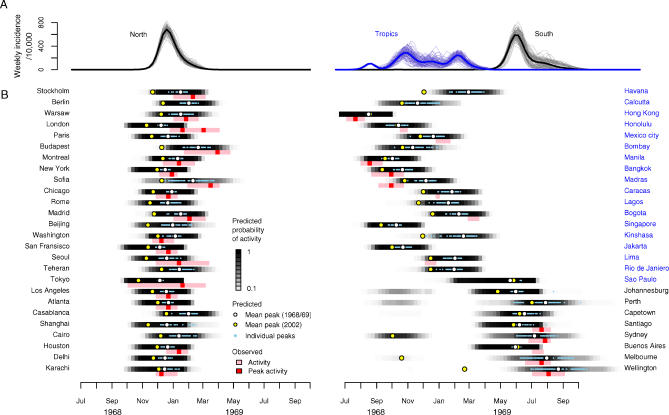
Predicted and Observed Times of Influenza Activity and Epidemic Peaks in 1968/9 (A) Predicted combined incidence using baseline model assumptions (bold lines show mean incidence). (B) Observed and predicted times in individual cities. Peak times from individual simulation runs and mean peak times with 1968/9 data are shown as blue and white dots, respectively. Mean peak times that would have occurred with 2002 travel patterns are shown as yellow dots. Predictions are based on 100 simulation runs. Influenza activity was defined as at least one new symptomatic case per 100,000 people in a given week.

The model showed good agreement with data from the 1968/9 pandemic, with observed epidemic peaks almost always occurring at times when the model predicted a very high probability of influenza activity (Figure 3). Observed and predicted times of epidemic peaks differed, on average, by 31 d. There were, however, some anomalies: the first epidemic peaks occurred much later than might have been expected in London and Tokyo, and somewhat earlier than predicted in Manila and Madras.

Despite large variation in the timing of predicted epidemic peaks in individual cities between simulation runs, the overall course of the pandemic was quite predictable (Figure 3A), although there was markedly more between-run variability in the tropics and the south than in the north. The roughly ten-fold increase in air traffic since 1968 causes epidemics in most cities to peak between 1 and 2 mo earlier than they would have done in 1968 (in some southern hemisphere cities the epidemic peaks 1 y earlier) and substantially reduces variation between simulation runs (Figure 3B). The model reproduced another interesting aspect of influenza epidemiology: the tendency for peak periods of influenza activity in the tropics to shift with latitude, so that in the northern tropics they are closer to countries north of the tropics, while the southern tropics tend to be more closely aligned with countries south of the tropics [24]. This occurs despite the fact that the model has no explicit assumptions about seasonality for cities in the tropics; the behaviour arises only as a result of the strength of transport connections between different regions. It is also notable that the pandemic starts early enough to allow some probability of influenza activity in the south during the end of the flu season in 1968. Despite this, predicted epidemic peaks (the weeks with the greatest number of reported cases in each location) still occur in 1969 in the south.

**Figure 4:**
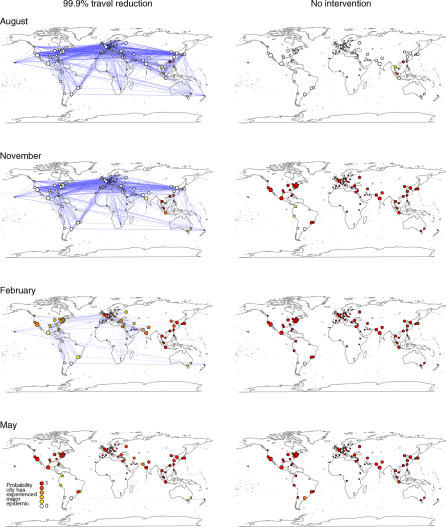
Time Course of a Pandemic with and without an Intervention to Suspend 99.9% of Air Travel from Affected Cities Maps show the extent of epidemic spread and average impact on aviation network (taken from 100 simulation runs) 2, 5, 8, and 11 mo after the first cases on 1 June. The intervention is made after 100 cases in each city (or 1,000 cases for Hong Kong, the city of origin). Blue lines represent flights, with darker blues representing greater mean weekly passenger numbers (after accounting for interventions to suspend travel and averaging over all simulation runs). Flights are not shown when travel restrictions have been imposed by the given time in more than 95% of simulation runs. Area of circles is proportional to city population size, and shading indicates the probability of each city having experienced a major epidemic (greater than one case per 10,000 people per day) by the given time.

**Figure 5:**
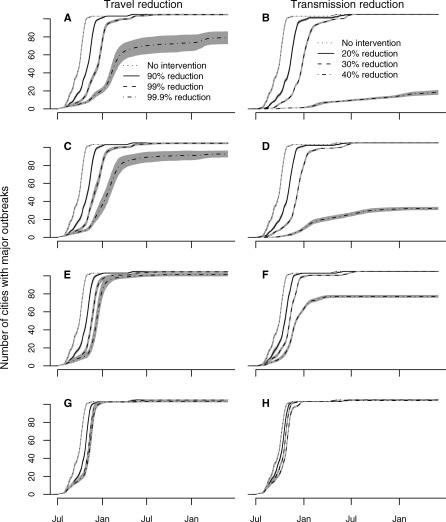
Impact of Interventions and Implementation Delays on Rate and Extent of Spread Effect of reducing travel (A, C, E, and G) and transmission (B, D, F, and H) on the timing of major outbreaks (≥ 1 case per 10,000 per day) in 105 cities using contemporary transport and demographic data and baseline parameters. Transmission reductions are imposed in each city after one case in the given city (A and B); 100 cases (C and D); 1,000 cases (E and F); and 10,000 cases (G and H). Interventions in the originating city (Hong Kong) occur after 1,000 cases (A–F) or 10,000 cases (G and H). Lines and shaded regions show means of 100 simulation runs and ± standard deviation, respectively. In all cases, *R*_0,max_ = 3. and the proportion of the population initially susceptible is 0.6.

**Figure 6:**
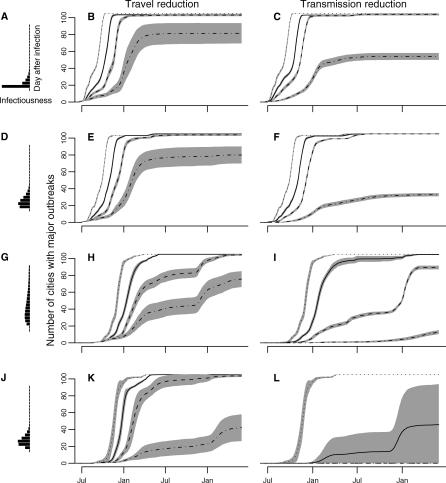
Impact of Interventions: Sensitivity to Disease Assumptions Impact of interventions under different assumptions about how the probability of being infectious and the degree of infectiousness varies with time since infection (A–I) and with latitude (J–L). Interventions, key, and other details are as in Figure 4, except all interventions occur after 1,000 cases in Hong Kong, and 100 cases elsewhere. (A–C) Variable infectiousness (baseline daily progression and recovery probabilities, but degree of infectiousness declines sharply after day 1 since infection). Mean serial interval = 2.6 d; mean infectious period = 3.0 d; mean latent period = 1.9 d. (D–F) Reduced latent period (constant infectiousness): Mean serial interval = 3.8 d; mean infectious period = 3.4 d; mean latent period = 1.2 d. (G–I) Extended infectious period (constant infectiousness): Mean serial interval = 8.6 d; mean infectious period = 3.9 d; mean latent period = 5 d. (J–L) Baseline parameters (constant infectiousness), but transmission in the tropics set to the mean of that in the temperate region. (A, D, G, and J) Proportion of secondary transmission that occurs 0–20 d after infection. In all cases, no transmission occurs on day 0 and *R*_0,max_ = 3.

When we used the model to evaluate interventions using contemporary air travel and demographic data, we found that travel restrictions to and from affected cities would slow epidemic spread, but unless almost all air travel from affected cities (i.e., greater than 99%) was suspended, the potential for delaying the pandemic was limited (Figures 4–6 and Table 1). Even when 99.9% of air traffic was suspended, most cities had a low probability of ultimately escaping the pandemic (Figure 4), and delays large enough to be of clinical significance (6 mo or more) were common only if interventions were made after the first few cases (Figure 5). Interventions that reduced transmission could typically lead to more pronounced delays (Figures 5 and 6 and Table 1), although only when *R*_t_ was reduced to slightly above one were these sufficient to delay epidemics until the next influenza season. These findings were not highly sensitive to assumptions about initial susceptibility and transmissibility (Table 1).

**Table 1:**
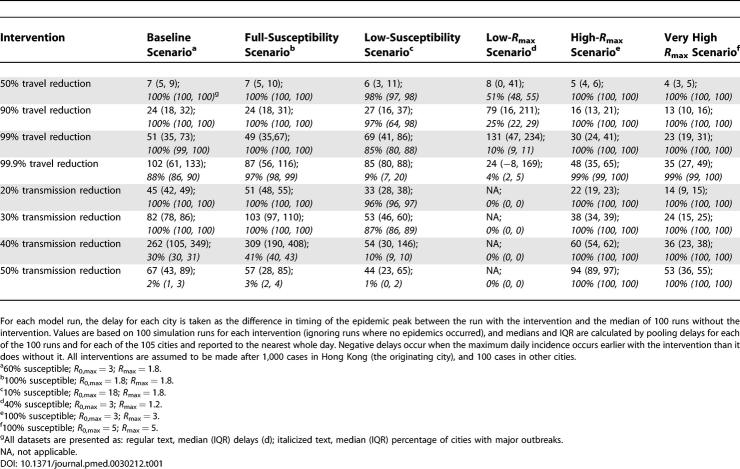
Median Delays in Epidemic Peak for Diverse Interventions and Percentage of Cities Experiencing Major Epidemics

Decreasing the number initially susceptible (while holding *R*_max_ constant) has two opposing effects (Table 1). First, within cities the time between seeding with influenza cases and the epidemic peak decreases. This is because the initial epidemic growth rate is unaffected, but each new case represents a greater proportional reduction in the susceptibles and causes a greater reduction in *R*_t_ (the epidemic peaks when *R*_t_ = 1). Conversely, between-city dynamics are slowed because there are fewer infectious people to spread the disease. Which effect dominates varies between cities; those affected at the start of the pandemic tend to experience peak activity earlier when there are fewer initial susceptibles; for the rest it usually occurs later. Although travel restriction always reduces the rate of spread between cities, under most scenarios so many people become infected that even near-total restriction has remarkably little effect. However, for a given *R*_max_, the smaller the number of susceptibles the greater the impact of this intervention. For example, when 90% of the population are initially immune, the most extreme travel restrictions can be quite effective in preventing international spread. Conversely, reducing transmission has the greatest effect on impeding international spread when (for a given *R*_max_) more people are susceptible. The large delays and reductions in the number of affected cities result from two effects acting in the same direction: The reduced *R*_t_ slows the epidemic within each city (delaying epidemic peaks), and the reduced total number of cases reduces the rate of spread between cities. Larger reductions in transmission led, in extreme cases, to smaller delays in epidemic peaks (Table 1). This happened only when *R*_t_ was reduced to below one, causing the epidemic decline to begin immediately; the peak therefore occurred at the time of the intervention, earlier than it would have done with a less effective intervention. Under such circumstances the time of the epidemic peak is not a good measure for fully evaluating local control measures.

Previous influenza modelling work has used both square and sine wave seasonal forcing terms [14,25]. We found that the outcomes of interventions were not highly sensitive to the precise assumptions made. The delays in the timing of epidemic peaks depended only to a limited extent on the city in which the pandemic started and to a somewhat greater extent on the date of release (Table 2), with larger delays more likely when the first cases occurred towards the end of the influenza season in the place of origin. Results were, however, highly sensitive to the timing of the intervention (Figure 5). Large delays in the timing of epidemic peaks and the prevention of epidemics in a large number of locations could be achieved with the most extreme interventions, but only when they were made sufficiently early. However, making the interventions after fewer than 1,000 cases in the place of origin had minimal additional benefit in slowing pandemic spread. Similarly, preemptive travel restrictions had no advantage over interventions made after one case in affected cities (Figure 5A and 5B).

**Table 2:**
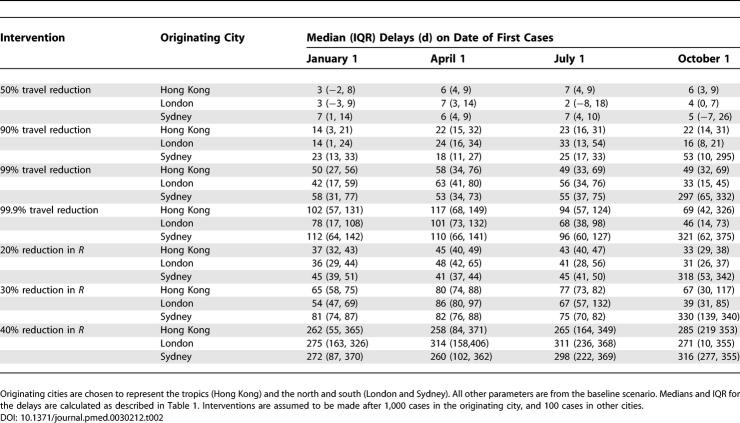
Median Delays in Timing of Epidemic Peaks with Different Dates and Locations for the Start of the Pandemic

The course of infection with a future pandemic influenza virus might differ in important ways from our baseline assumptions, and could be quite unlike typical interpandemic influenza. We therefore assessed the robustness of our conclusions to the assumed latent and infectious periods. We found that assuming a greater degree of infectiousness early in the course of infection (reducing the serial interval from 4.2 to 2.6 d, as suggested by recent analysis of household influenza transmission data [11,26]) did not substantially alter the conclusions about the value of the interventions (Figure 6A–6C) compared with the baseline scenario (Figure 5C and 5D), although if this assumption was used when fitting the model to the 1968/9 data the estimated value of *R*_max_ was reduced from about 1.8 to 1.5. Conclusions were also robust to moderate variation in the distribution of the latent period (Figure 6D–6F). If, however, the virus behaved more like the SARS coronavirus, with extended latent and infectious periods (Figure 6G–6I), a greatly delayed rate of global spread could be expected, giving more chance of delaying epidemics until the next influenza season. In this case, smaller reductions in travel and transmission can achieve clinically significant delays (6 mo or more) in epidemic take-off in many cities. Assuming reduced transmission in the tropics (Figure 6J–6L) also led to a substantial reduction in the rate of global dissemination. Under this scenario much smaller reductions in transmission would be sufficient to greatly reduce the chance of a pandemic; this happens because the lower transmission in the tropics (where the virus is assumed to originate) means that a further transmission reduction of just 21% would be sufficient to make sustained spread impossible in this region.

## Discussion

The relative ineffectiveness of travel restrictions for controlling pandemic influenza is a consequence of the rapid initial rate of growth of the epidemic in each city and the large number of people infected. For example, with a serial interval of 3 d, ignoring depletion of susceptibles, an *R*_t_ of two would cause a 128-fold increase in new cases within 21 d (128 = 2^21/3^). This means that if travel from the first affected city was restricted to 1/128 of its former value on (and after) day 1, there would be approximately the same number of influenza cases leaving the city on day 21 + *t* as there would have been on day *t* had there been no intervention; even such an extreme intervention would therefore buy only about 3 wk. The highly connected nature of the air travel network prevents such minor delays between pairs of cities combining into substantial delays over the whole network.

Hufnagel et al. [20] used a related model to study the global spread of SARS. Although this model differed in important respects from the one used here (the implicit assumptions that air travel frequency varies with neither infection state nor country would not be tenable in the context of pandemic influenza), the conclusion that “remarkable success [in SARS epidemic control] is guaranteed if the largest cities are isolated in response to an outbreak” might, at first sight, be thought to apply equally to influenza. In fact, pandemic influenza is expected to have a much shorter serial interval than SARS, and delays in international spread that could be achieved by restricting almost all travel would be far more modest. Even if 99.9% of all travel could be stopped, epidemics in most cities would be delayed by no more than 4 mo. Moreover, the conclusion that a policy of isolating only the largest cities would guarantee success implicitly assumes that closing major airports would cause infected individuals who would have travelled through them to abandon their journeys rather than seek alternative routes, and that disease spread by routes other than air travel can be ignored without substantially altering the conclusions. This seems rather implausible, and for these reasons we think that the conclusions of Hufnagel and colleagues, while of undoubted theoretical interest, would be misleading if taken too literally.

Large and important uncertainties abound in influenza epidemiology: We do not know whether or not a significant proportion of transmission occurs before the onset of symptoms or whether subclinical infections are an important source of transmission, and we know very little about the determinants of seasonality [24,27,28]. In evaluating the potential to delay the spread of influenza by restricting travel and reducing transmission, we have systematically adopted optimistic assumptions, chosen to give the interventions the greatest chance of success. Thus we have assumed that seasonal effects are important (delaying the rate of spread outside the influenza season), and that asymptomatic cases do not contribute to transmission (minimizing the numbers capable of spreading the virus, and maximising the chance of detecting them); we have ignored travel that is not by air and not between major airports; and we have ignored the possibility of transmission during flights themselves. Despite these optimistic assumptions we found that even large and widely enforced travel restrictions would usually delay epidemic peaks by only a few days; to have a major impact, restrictions would have to be almost total and almost instantaneous. Only if a pandemic strain were considerably less transmissible, or had a considerably longer serial interval than influenza strains seen in the past, or if very few people were initially susceptible, would such measures be likely to have an important impact on the rate of pandemic spread. Local control measures able to reduce influenza transmission were found to have greater potential for reducing the rate of global spread (they could also substantially reduce the total number of cases, although an evaluation of this benefit is beyond the scope of this paper). Under most plausible scenarios, however, delays would still fall far short of those required to produce large quantities of vaccine unless they were implemented early and able to reduce *R*_t_ to close to one. Elsewhere it has been shown that airport entry screening would be unlikely to detect more than 10% of passengers latently infected with influenza when boarding [29]. The results in this paper show that such an intervention would have a negligible impact on the course of a pandemic once it was underway.

The results also raise interesting questions about the importance of seasonality in influenza transmission. The evidence for strong seasonal effects in temperate regions found here with 1968/9 data is supported by a recent analysis of interpandemic influenza [30]. However, it is not clear how important such seasonal effects have been in previous pandemics, nor is it clear why a much better model fit should be obtained when transmission in the tropics is assumed to be the maximum (rather than the mean) of that in temperate regions. Indeed, a fuller understanding of the determinants of seasonal effects and their variation with latitude remains one of the outstanding problems of influenza epidemiology [24,27,28].

Recent models of pandemic influenza have accounted for household and social contact patterns [10,11]. While such details are needed for evaluating the possibility of containment at source, they would not be expected to affect the broad conclusions presented here. However, for a given *R*_0_, assuming nonhomogeneous local mixing patterns would result in a somewhat reduced attack rate and rate of spread within each city, causing a slight decrease in the rate of global spread. For this reason, estimates of *R*_max_ based on fitting models that assume homogeneous local mixing to pandemic data may underestimate the true value.

A new pandemic strain might not show the same pattern of seasonality as in 1968/9 and could potentially have greater transmissibility than strains seen previously. Both SARS and smallpox transmission can be greatly amplified by nosocomial spread [31,32]; a similar amplification effect could occur with an unusually virulent influenza virus that led to many hospitalisations. In these more pessimistic scenarios, even more heroic efforts would be required to have any chance of significantly delaying the virus's spread by restricting travel. The results here suggest that resources might be better directed at reducing transmission locally and at attempting to control outbreaks during the earliest stages of sustained human-to-human spread, when movement restrictions are likely to be a more valuable containment measure [10,11]

## Supporting Information

Protocol S1 Detailed Description of the Model (65 KB DOC)

**Editors' Summary**

**Background.**

Most people who get influenza (flu) recover quickly, although it can cause serious illness and death, most often in the elderly. Sometimes a new type of flu virus appears that is much more likely to kill. This happened, for example, in 1918, when a worldwide flu pandemic killed between 20 million and 100 million people. Recently, there have been concerns about a flu virus that affects birds, and often kills them. At present the virus does not pass easily from birds to humans, and it does not seem to pass from one human to another. However, the fear is that this virus might change and that human-to-human infection could then be possible. Should all this happen, the changed virus would be a major threat to human health. With current technology, it would take several months to produce enough vaccine for even a small proportion of the world's population. By that time, it would probably be too late; the virus would already have spread to most parts of the world. It is therefore important for health authorities to consider all the methods that might control the spread of the virus. With the increase in international travel that has taken place, the virus could spread more quickly than in previous worldwide pandemics. Restrictions on international travel might, therefore, be considered necessary, particularly travel by air.

**Why Was This Study Done?**

It is important to estimate how useful restrictions on air travel might be in controlling the spread of a flu virus. Travel restrictions are usually unpopular and could themselves be harmful, and, if they are not effective, resources could be wasted on enforcing them.

**What Did the Researchers Do and Find?**

This research involved mathematical modelling. In other words, complex calculations were done using information that is already available about how flu viruses spread, particularly information recorded during a worldwide flu outbreak in 1968–1969. Using this information, virtual experiments were carried out by simulating worldwide outbreaks on a computer. The researchers looked at how the virus might spread from one city to another and how travel restrictions might reduce the rate of spread. Their calculations allowed for such factors as the time of the year, the number of air passengers who might travel between the cities, and the fact that some people are more resistant to infection than others. From the use of their mathematical model, the researchers concluded that restrictions on air travel would achieve very little. This is probably because, compared with some other viruses, the flu virus is transmitted from one person to another very quickly and affects many people. Once a major outbreak was under way, banning flights from affected cities would be effective at significantly delaying worldwide spread only if almost all travel between cities could be stopped almost as soon as an outbreak was detected in each city. It would be more effective to take other measures that would control the spread of the virus locally. These measures could include use of vaccines and antiviral drugs if they were available and effective against the virus.

**Additional Information**

Please access these Web sites via the online version of this summary at http://dx.doi.org/10.1371/journal.pmed.0030212.

•  Fact sheets are available about various aspects of flu from the Web site of the World Health Organization, which takes a global overview of the impact of the infection

Many health Web sites aimed at patients provide basic information about flu.

• US National Institute of Allergy and Infectious Diseases page about flu

• National Institute of Allergy and Infectious Diseases fact sheet about cold and flu symptoms

• US Centers for Disease Control and Prevention page about flu

• The Journal of the American Medical Association's patient page about influenza

• Page on flu from BBC Health

• Information about pandemic influenza from The Health Protection Agency

## Abbreviations

IQR: interquartile range
SARS: severe acute respiratory syndrome
SSQ: sum of squared deviations
