# Supplementary material for: Delaying the International Spread of Pandemic Influenza
Source: PLoS Med. 2006 May 2;3(6):e212. doi: 10.1371/journal.pmed.0030212 (PMC1450020; doi:10.1371/journal.pmed.0030212)
Supplement: Supplementary file 1 [file pmed.0030212.sd001.doc]

**Supporting Information**

The model has the following variables:

$x_{i}(t)$ is the number of susceptible individuals in city *i* at time *t* where *t*∈{0,1,2,…}.

$u_{i}(\tau,t)$is the number of latently infected individuals in city *i* at time *t* who were infected on day *t*-*τ,* where *τ*∈{0,1,2,…}.

$y_{i}(\tau,t)$is the number of infectious individuals in city *i* at time *t* who were infected on day *t*-*τ*.

Probabilities of progression from latent to infectious and infectious to recovered (and immune) are specified by two probability distributions:

γ(*τ*) gives the probability that a latently infected person infected *τ* days previously becomes infectious by day *τ*+1.

δ(*τ*) gives the probability that an infectious person infected *τ* days previously becomes immune by day *τ*+1

α(*τ*) is a factor to account for variation in the probability of transmission from an infectious person with time since infection, *τ*.

In accordance with previous work (13), for the baseline scenario we take

γ(0)=0.3, γ(1)=0.71, γ(2)=1.0 ,γ(*τ*)=0 for *τ*>2,

{δ(0),δ(1),...,δ(8)}={0.0, 0.1, 0.19, 0.34, 0.44, 0.50, 0.60, 0.83, 1.0} , δ(*τ*)=0 for *τ*>8 and α(*τ*)=1 for all*τ*. This gives a serial interval of 4.2 days.

$n_{i}$is the population size of city *i*

$\sigma_{ij}$is the daily population flow from city *i* to city *j*.

We define the following random variables:

$I_{i}(t)$is the number who become infected in city *i* at the end of time step *t*

$P_{i}(\tau,t)$is the number of latently infected people who remain in city *i* who were infected at time t-*τ* and who progress to become infectious at the end of time step *t.*

$R_{i}(\tau,t)$is the number of infectious people in city *i* who were infected at time t-*τ* who progress to become immune at the end of time step *t*.

$F_{ij}(\tau,t)$is the number of latently infected people in city *i* who were infected at time t-*τ* who move to city *j* at the end of time step *t.*

$G_{ij}(\tau,t)$is the number of the latently infected people in city *i* who were infected at time *t*-*τ* who move to city *j* at the end of time step *t* and who are infectious on arrival at time *t*+1 *.*

Distributions are given by:

$$\begin{matrix} F_{ij}(\tau,t)\sim\mathrm{Bin}(\varphi_{ij}(t)\sigma_{ij}/n_{i},u_{i}(\tau,t)) \\ G_{ij}(\tau,t)\sim\mathrm{Bin}(\gamma(\tau),F_{ij}(\tau,t)) \\ I_{i}(t)\sim\mathrm{Bin}(\lambda_{i}(t),x_{i}(t)) \\ P_{i}(\tau,t)\sim\mathrm{Bin}(\gamma(\tau),u_{i}(\tau,t)-\sum_{j} F_{ij}(\tau,t) \\ R_{i}(\tau,t)\sim\mathrm{Bin}(\delta(\tau),y_{i}(\tau,t)) \\ \end{matrix}$$

Here $\varphi_{ij}(t)$ represents the proportional reduction in population flow from city *i* to *j* at time *t* resulting from control measures.

$\lambda_{i}(t)$is the probability that a susceptible person in city *i* becomes infected by time *t*+1. In general it will depend on the number of infectious people in the city, the distribution of times since they were infected, seasonal factors, and control measures in place. If *λ/n_i_* is the probability of an effective (i.e. potentially infectious) contact between two individuals in city *i* in a single time step, then the probability that a susceptible person becomes infected by one of the *y* infected people is $1-(1-\lambda/n_{i})^{y}$. This is approximately *λy/n_i_* when *λ/n_i_* is small, as it will be here. We therefore put$\lambda_{i}(t)=\sum_{\tau} \lambda y_{i}(\tau,t)\alpha(\tau)\theta_{i}(t)s_{i}(t)/n_{i}$, where $\theta_{i}(t)$represents the proportional reduction in the transmission in city *i* at time *t* as a result of control measures or behaviour modification, and $s_{i}(t)$represents the seasonal modulation of transmissibility in city *i* at time *t*.

The model is updated at each time step according to the following equations:

$$\begin{matrix} x_{i}(t+1)=x_{i}(t)-I_{i}(t) \\ u_{i}(0,t+1)=I_{i}(t) \\ u_{i}(\tau+1,t+1)=u_{i}(\tau,t)-\sum_{j} F_{ij}(\tau,t)+\sum_{j} F_{ji}(\tau,t)-\sum_{j} G_{ij}(\tau,t)-P_{i}(\tau,t) \\ y_{i}(\tau+1,t+1)=y_{i}(\tau,t)-R_{i}(\tau,t)+P_{i}(\tau,t)+\sum_{j} G_{ji}(\tau,t) \end{matrix}$$

If seasonality varies sinusoidally we can put $s_{i}(t)=[(1-R_{0,\min}/R_{0,\max})\sin(2\pi(t-t_{\max,i})/365+\pi/2)+1+R_{0,\min}/R_{0,\max}]/2$ for cities outside the tropics, where $t_{\max,i}$is the day where transmissibility is maximum in each hemisphere. For cities in the north we take this to be day 356, and for those in the south day 173. In this formulation we set *λ* equal to $R_{0,\max}$/2.95, where 2.95 is the mean number of days of infectiousness for the parameters used here. In the tropics $s_{i}(t)=1$. Seasonality can be represented using a step function in a similar manner. In the tropics, the maximum value for the north and south were assumed to apply throughout the year.

We also have $y_{i}(0,t)=0$ for all *t* and epidemics are initialised by setting $u_{i}(0,0)$to 20 in the city, *i*, where the pandemic is assumed to originate.

**Sensitivity analysis**

To account for the possibility that the degree of infectiousness may decline sharply with the time since a case is first infectious [25, 26] we performed a sensitivity analysis (figure 6A-C) where we took {*α* (0), *α* (1),..., *α* (8)}={0.0, 1.0, 0.1, 0.05, 0.04, 0.03, 0.02, 0.01, 0}and *α* (*τ*)=0 for *τ*>8, giving a serial interval of 2.6 days.

Further sensitivity analyses were performed to investigate the effects of (i) a shorter latent period (figure 6D-F) and (ii) a greatly extended infectious period (figure 6G-I). This was done by setting (i) γ(0)=0.8, γ(1)=1, and γ(*τ*)=0 for *τ*>1 and (ii) γ(0)= 0, γ(1..16)= 0.25, γ(17)= 1, γ(*τ*)=0 for *τ*>17, and δ(0..1)=0, δ(2..19)=0.25, δ(20)=0, with *α* (*τ*)=1 for all *τ* in both cases. Unless specified otherwise, in all sensitivity analysis *λ* was adjusted to maintain the same effective reproduction number at the start of the pandemic, and all other parameters were set to baseline values.
